# Real-world evidence to support health technology assessment and payer decision making: is it now or never?

**DOI:** 10.1017/S0266462325000145

**Published:** 2025-03-31

**Authors:** Linda A. Murphy, Ron Akehurst, David Cunningham, Gérard de Pouvourville, Oriol Solà-Morales

**Affiliations:** 1 Lumanity Inc, Sheffield, UK; 2 Independent Consultant, Surrey, UK; 3 ESSEC Business School, Cergy, France; 4 Fundació HiTT, Barcelona, Spain

**Keywords:** clinical need, data governance, guidance, health technology assessment, payers, population health, real-world data, real-world evidence

## Abstract

**Objectives:**

The aim of this policy article is twofold: (i) to provide a summary and update of recent important policy developments, in particular relevant guidance on the use of real-world data/real-world evidence (RWD/RWE) by health technology assessment (HTA) bodies and (ii) to set out our policy recommendations on how the different elements of an “RWE framework” we have previously developed could support, further enhance and facilitate the use of RWE for HTA purposes and by HTA bodies and payers.

**Methods:**

We undertook a targeted review and analysis of recent important policy developments. The aim was to build on our recommendations from previous work on the “RWE Framework,” and consider how the relevant tools from our Framework can further enhance and facilitate the use of RWE for HTA purposes and by HTA bodies/payers.

**Results:**

We provide eight conditions that we argue would, in combination, constitute the optimal use and acceptance of RWD/RWE for HTA. We believe that, should the eight conditions hold, RWD/RWE would enable more efficient access to medicines and healthcare technologies for patients.

**Conclusions:**

High-quality, fit-for-purpose RWD/RWE can and should be used more frequently in HTA. Multi-stakeholder and cross-geography collaborative partnerships are needed to align on best practices to optimize the evidence that needs to be generated to satisfy all stakeholders’ needs.

## Introduction and objectives

There have been increasing calls for greater use of real-world data and real-world evidence (RWD/RWE), to enable the achievement of better health outcomes by faster provision of effective medicines to patients in need (1). This is driven by the desire to understand how technologies work in clinical practice once the marketing authorization (MA) is granted by the relevant regulatory authorities. This MA traditionally relies primarily on the evidence generated by randomized clinical trials (RCTs) (2), deemed the gold standard for estimating the efficacy provided by a new treatment in given circumstances and in a selected population, as well as understanding its safety. However, RCTs also have their weaknesses, with many questions that they are not able to answer even in the context of relative efficacy (3). Thus, regulators have taken an active role in issuing guidance on how and when RWD can be used to support regulatory decision making (see, for instance, FDA (4), and their relevant documentation found in the Center for Biologics Evaluation and Research & Center for Drug Evaluation and Research Real-World Evidence webpage (5)). In Europe, in particular, RWD is traditionally used by regulatory bodies to collect safety information on technologies with an MA and, in some cases, to provide evidence of efficacy (6). The European Data Analysis and Real-World Interrogation Network (7) (DARWIN EU) can be a key milestone within the regulatory realm; its stated objective is to deliver RWE from across Europe on diseases, populations, and the uses and performance of medicines to support regulatory decision making. It is expected to deliver 150 RWE studies annually since the first pilot studies in 2022, and circa 380 studies over the first years (8).

The subsequent assessment of such technologies for the pricing and reimbursement decisions taken by individual countries and/or payers is also frequently based on RCTs (2). However, when we embarked on this project approximately 6 years ago, there was very limited guidance on the use of RWD for health technology assessment (HTA), pricing, and reimbursement decisions, leading to a patchy use of RWE to support HTA decision making more generally (9–11). More recently, RWD/RWE use has been deemed as suboptimal during this process (2;3). While we have not found a definition of what could be considered as optimal use, one of the key premises for our work was that important aspects of RWE use in the assessment of medicines by HTA agencies and payers were contentious and also variable between jurisdictions, and that the path of new pharmaceuticals to patients could be made quicker and more efficient were that not to be so (3). For this reason, an objective of ours was to develop a “RWE Framework,” aiming to provide solutions and tools to support, improve, and optimize the use of RWE for HTA purposes. One specific aim was to provide a structure for a repository of experience in using RWD, which would guide manufacturers and assessment agencies alike. This “RWE Framework” was published as a supplement of five papers in 2023 (1;3;12;13;14) and we summarize its key elements in this policy article. To develop the RWE Framework, we took a pragmatic and iterative approach enhanced by case studies and key stakeholder engagement to gather and synthesise evidence in an efficient way and to answer some key questions related to our topics. We aimed to be systematic and transparent in our approach and to be robust while efficient. All the details can be found in Murphy et al. (10). Building from this RWE Framework, the key objective of this policy paper is further dissemination and engagement, hoping it will support the advancement in the use of RWD/RWE for HTA decision making. For this purpose, we first provide a summary of recent important policy developments, including relevant guidance on the use of RWD/RWE by HTA bodies. This includes work since the supplement was produced. We then compare this guidance with the relevant tools from our Framework. Second, we set out our policy recommendations on how the different elements of the “RWE framework” could support, further enhance, and facilitate the use of RWE for HTA purposes and by HTA bodies.

Broadly speaking, in the five supplement papers, there are two key tools underpinning the Framework. First, an RWD/RWE taxonomy or road map, offering a structured classification of decision types using RWE, around which evidence can be assembled in an actively managed or curated source. The taxonomy, described in Murphy et al. (1), includes a list of RWD sources (e.g., EHRs, registries) and questions (e.g., estimating resource use), which could be answered via these sources, that is, linking questions to sources − termed “pairings” (1).

Second, a data governance (DG) checklist, described in Solà-Morales et al. (14) recommends a set of international standards of how the acceptability of RWD/RWE should be assessed, which could then benefit RWD users, such as public/private researchers, data managers, and HTA bodies (14).

The supplement includes three other interrelated papers, which also form the basis of the two key tools: a paper proposing a structured classification of decision types using RWE, around which evidence can be assembled in a curated source (RWD/RWE taxonomy) and thus facilitate judgments on when evidence is “good enough” (3); a paper on case studies (countries), assessing how RWE has been used to support decision making in practice, identify the hurdles to the acceptance of RWE, and suggesting directions toward a more effective use (12); and a detailed explanation of the methodology used to conduct the work (13).

Akehurst et al. (3) offer the following revised definition for RWD and RWE, building from numerous definitions identified by literature reviews and incorporating how its elicitation is evolving:

**RWD**: data collected typically in a noninterventional setting (thus not typically a randomized controlled trial [RCT]) so that the data collected reflect everyday activities relevant to health, irrespective of the environment in which they are harvested or the mechanism of collection. Randomization, per se, does not make data non-real, as pragmatic trials generate RWD. The latter are “interventional” but aim to include patient populations that would be found in everyday clinical practice.

**RWE**: evidence derived through analysis and interpretation of RWD (or RWD plus RCT data) using best practice methods.

More positively, national and international initiatives with the overall objective of improving the use of RWD for HTA have been introduced over the last few years, and we discuss some of them in this article. While they are still very limited in number, importantly, they have been developed via transparent and multi-stakeholder discussions, leading to agreed guidance on RWD/RWE and real-world studies both for those in industry generating the models and for those in HTA bodies assessing the quality of the evidence and the studies. There is evidence showing increasing RWD use for HTA purposes; for instance, Aitken et al. (15) show how the proportion of HTA reports including RWE as part of the submission has risen from 6 percent in 2011 (i.e., 43 out of 716 HTAs included in the analysis for 2011 had RWE, while the rest did not use RWE) to 39 percent in 2021 (706 out of 1,809 HTAs had RWE that year). However, and importantly, the impact of the RWD on the actual decision is not discussed.

We believe the use of RWE in HTA will continue to increase. How much and with what impact, is yet to be seen, but it is foreseeable there would still be differences in acceptance and use across countries.

This policy article is structured as follows. First, we summarize briefly some of the challenges with the use of RWD for HTA purposes, identified from our previous work and the additional literature reviewed subsequently. Second, we discuss how some recent individual guidance by HTA bodies on RWE use, entailing multi-stakeholder participation and collaboration, compare across each other and with the two key tools from our RWE framework – with a high level of alignment. Third, we consider an “ideal” situation in terms of the acceptance and use of RWD/RWE in HTA decision making, where the challenges identified previously could be overcome. Fourth, we set out our recommendations on how the different elements of the RWE framework could help achieve this optimal situation. This is followed by a discussion section, and the paper concludes with two final reflections.

## Challenges in the use of RWD for HTA, pricing, and reimbursement

Building on our previous work, we have identified the following four key (and interrelated) challenges that help explain the limited and patchy use of RWD/RWE during pricing, reimbursement, and HTA processes.

### Key challenge 1: moving from the general to the particular when accepting RWE

A central conclusion of our published reviews was that general statements about the optimal use of RWE, although valuable, would always have limitations because problems were almost invariably particular to the use of particular data sources, answering particular questions (with particular associated consequences of risk of a biased decision). The second issue was that lack of clarity over DG, the interaction between the selection and use of data and the rules governing access to it, hampered provision and use of helpful data (see key challenge 3).

In parallel, the lack of a collectively agreed, established and common terminology that discusses the questions that RWE can answer also hinders RWE acceptability within HTA decision making. Publications that provide general guidance on the use of RWE will not, on their own, solve this problem. This vast amount of information can be challenging to navigate for the practitioner (i.e., the person using the information) and HTA decision makers, which can hinder timely solutions, particularly when a timely answer is needed.

### Key challenge 2: practical implementation of RWD/RWE and the need to define “fit for purpose”

There is also the uncertainty surrounding what could be deemed “fit for purpose” evidence, with two questions possibly underpinning this uncertainty:

1. Are the requirements being asked for feasible?
2. Is there a communication issue between the academics/RWD experts and the policy makers (i.e., those who need to pass on the laws that will dictate if and how RWD/RWE is used in practice)?

Early dialogue, and opportunity for continuous dialogue, between both parties (HTA body/assessor and manufacturer) is important, to agree on what is “fit for purpose,” among other things. There is a need to move from “academic perfection” to practical and pragmatic implementation for RWE to have a positive effect on the efficiency of the HTA process. For instance, early engagement (e.g., scientific advice) combined with guidelines (as we comment below, and importantly, including DG) could well be the practical way forward, allowing for pragmatism and reasonable predictability.

### Key challenge 3: DG and transparency

DG can be defined as a strategy for the overall management of the usability, availability, integrity, quality, and security of data to ensure its maximum potential (16). The lack of clarity over DG primarily in the HTA setting, the interaction between selection and use of data and the rules governing access to it, hampers provision and use of helpful data (3); data sharing is also a large concern, especially considering evolving data protection regulations (14). These effects are exacerbated when there is no adequate data infrastructure or transparency in study design. The vast amount of available RWD and the emerging opportunity to use RWE in decision making require thoughtful and appropriate governance, facilitating the appropriate collection of evidence, ensuring the data are of adequate quality, and making the most out of the opportunity it presents to improve patient care.

Lack of transparency in two further interlinked aspects were identified: (i) how data sources were chosen and used by companies submitting to decision-making bodies and (ii) how those analyses were received and used by the bodies to reach their decisions (3).

### Key challenge 4: the challenges faced by HTA decision makers that remain unresolved

Additional unresolved challenges have been identified. These have been validated by our Advisory Board with HTA stakeholders (see Akehurst et al. (3) and Murphy et al. (13) for more details), and highlighted recently by Besley et al. (2). These include:

- methodology, including transferability/generalizability of results, or treatment effect estimates, and associated statistical methods
- role of RWE, with lack of consensus from HTA bodies on how and when to use RWE across a product lifecycle (in part could reflect a lack of technical expertise within HTA bodies), and lack of alignment of RWE requirements pre- and post-licensing (regulatory vs. HTA/Payer bodies)
- lack of collaboration across stakeholders.

## Overview of recent relevant national and international guidance and initiatives

Several pieces of guidance have recently been established that address some of the key challenges mentioned. We report on four of them at country/region level, where HTA bodies have published their own guideline (and checklists) on the use of RWD/RWE for their decision making (CADTH (17) also includes regulatory approval, NICE (18), AQuAS (19) in Catalonia, and HAS (20)). While there are many “official” guidelines, the four included here serve as illustrative examples. We note that all are widely discussed in the literature and in policy fora, and are from well-respected organizations. Importantly, the lack of transparency in the generation of RWD is cited frequently as an area of concern, and a key objective of the guidelines is to both increase the transparency of, and the trust in the data underpinning the real-world studies used for the assessment of treatment effects in particular. In addition, some countries have wider RWD/RWE strategies that go beyond HTA.

Table 1 provides a high-level summary on the stated key objective, and the potential users/targets of each of the four documents, plus the DG checklist from Solá-Morales et al. (14) for ease of comparison.

**Table 1.** Summary of objectives and potential users of HTA bodies’ guidance (15–18) and DG checklist from Solá-Morales et al. (9)

CADTH: Guidelines for reporting RWE	NICE RWE framework	AQuAS: Protocol for studies using RWD to generate quality evidence for HTA	HAS: Real-world studies for the assessment of medicinal products and medical devices	Solá-Morales et al data governance for RWD management: A proposal for a checklist to support decision making
Stated objective: lay the foundation for the use of RWE both for regulatory approval and HTA in Canada, facilitating its appraisal for supporting decision making. The guidance promotes standardization in reporting of RWE studies, built from reviewing the international experience, and establishing consensus on items to be included in the core reporting standards for Canadian RWE studies	Stated objective: living framework that will be updated periodically to reflect user feedback, learning from implementation including exemplar case studies, developments in real-world evidence methodology, and to extend its scope to include additional guidance on priority topics It includes best-practices for planning, conducting and reporting RWE studies to improve the quality and transparency of evidence	Stated objective: internal methodological guidelines for studies with RWD carried out by them, with the objective of standardizing their methods	Stated objective: support and assist the implementation of real-world studies relating to health products assessed by HAS assessment committees. It aims to provide practical benchmarks relating to methodological aspects to optimize the level of evidence of these studies and confidence in their results	Stated objective: propose recommendations for international standards of evaluating the acceptability of RWD governance practices.
Users: be those developing and reporting RWE to regulatory and HTA bodies as well as those who review and appraise the evidence	Users: those developing evidence to inform NICE guidance; also relevant to patients, those collecting data, and reviewers of evidence	Users: Internal use	Users: pharmaceutical companies and contract research organizations	Users: RWD/RWE users, such as public/private researchers, data managers, and HTA assessment bodies

These documents address the four general challenges highlighted above on the use of RWD/RWE for HTA decision making by:

- offering some “particular” recommendations across various domains, with the expectation that if the data/study meets them, they will be used. However, flexibility is important. HAS stresses that the guideline is not a binding document and should be seen as a methodology guide rather than as providing a “ready-made formula” that can be applied in all circumstances – it has to be applied responsibly by the manufacturers;
- providing guidance on what could be deemed as appropriate data for its purposes – while acknowledging that, NICE, for instance, states it does not set minimum acceptable standards for the quality of evidence;
- providing a bridge pulling together the views and expectations of the different organizations and associations involved in the HTA process about what is required, and expected, from RWD. These documents are based on solid academic foundations where available and have gone a step beyond by becoming official guidance.

These initiatives highlight that to have an impact, the process to achieve the final deliverable is as important as the guidance itself, with collaboration across all agents being imperative. There are numerous aspects to cover to ensure the RWD can be used for HTA purposes, and each agent should provide its relevant skills and expertise. No single stakeholder will be able to address all the challenges on their own.

Finally, there is close alignment across the five documents in terms of scope, and potential users with some broad agreement on methods/approaches to tackle the key challenges.

## RWD/RWE for HTA: an “ideal situation”

Table 2 provides eight conditions that would in combination constitute, at least in our view, the optimal use and acceptance of RWD/RWE for HTA. We believe that, should the eight conditions hold, RWD/RWE would facilitate making medicines and healthcare technologies accessible to patients.

**Table 2.** Outline of an “ideal situation” on use and acceptance of RWD/RWE for HTA

	Ideal situation
I	RWD/RWE is fit for the purpose for which it is used. Studies generating new RWE do so with a clear question in mind using sources and methods for analysis that are widely accepted as appropriate for the case in point. A decision pair of question and data source is at the forefront of any discussion of RWE acceptability. Existing RWD, for example data generated in clinical practice, is also leveraged for (unforeseen) secondary use, for example, for research
II	RWD is “fit for purpose” (i.e., meets the appropriate quality standards) to answer the questions being asked of it. The standards by which it is judged are appropriate for the task
III	There is a consistent adoption of a minimum threshold of data governance, which will increase confidence in the data
IV	There is clear terminology, with a shared language to help remove ambiguity and uncertainty, facilitating the conversation around RWE
V	There is a clear roadmap (i.e., labeling and categorization) of RWE information. A “bringing together” of key information from which we can continuously improve RWE use (pairings in practice, guidance, methods, etc.)
VI	There is improved transparency in RWE generation
VII	The specific challenges faced locally are known/clear and there are suggestions for solutions
VIII	Collaboration between RWE initiatives, and across stakeholders early on, is enhanced and supported

## Recommendations to support reaching an “ideal situation”

Based on the background work carried out to develop our RWE Framework, including the detailed external engagement process, and the additional work carried out for this policy paper, we believe that if the use of RWD is further enhanced and facilitated for the HTA decision-making process, this would support bringing effective new medicines to the patients that need them. The recent initiatives mentioned above align well with our aspirational “ideal situation,” and our RWE framework supports them. There have been calls for a repository of real-life examples and experiences in using RWD to support HTA and related decision making (either because the RWD/RWE has been used, or requested, by HTA bodies), as well as an appropriate, helpful structure for keeping and retrieving the material. We recommend using the taxonomy presented in Akehurst et al. (3) and Murphy et al. (1) as the basis of this “live” repository. Users will be able to find the information quicker, supported by the other elements of the framework, enabling them to:

1. identify the issues they will have to grapple with in using their data sources to answer their question;
2. easily identify relevant, high-quality methodological guidance;
3. review the experience of others faced with similar challenges in practice – how have the agencies receiving submissions tackling similar questions responded in the past.

Our second recommendation is that whatever shape or form the taxonomy in Murphy et al. (1) takes, a selected organization should set up a repository of real-life examples and experience in using RWD and actively manage and run it. This will allow for quality assurance measures to be incorporated. Moreover, the RWE framework could be leveraged to collaborate with the existing (and any future) EU initiatives, even if they are currently for regulatory decision making, to support the EU vision of reducing the fragmentation in European HTA processes.

The version of the taxonomy presented in Akehurst et al. (3) and Murphy et al. (1), is in, principle, “country-agnostic”; thus, we recommend that it is developed at the national level, so that its actual use would depend on the stage of “maturity” with RWD/RWE use. For instance, for countries without specific RWE guidance, or in the process of developing them, our work could help guide them. For those few countries with RWE guidelines, our work is complementary.

Ultimately, the way forward with the taxonomy should be balanced with the practicalities of who is ready, willing, and able to work on setting it up and its “active management”. For that purpose, we carried out a preliminary mapping exercise thinking about potential roles and responsibilities across the relevant organizations/stakeholders at the European and national level (HTA agencies, regulators, industry, payors/healthcare systems, policy/decision makers, and patients). This is shown in Table 3 with a brief rationale regarding their potential interest and benefits.

**Table 3.** Taxonomy – exploring the interest and role of stakeholder groups

	HTA agencies/bodies	Regulator	Industry	Payor/HCS	Policy/decision maker/healthcare practitioner	Patient group
Role^a^	E/C/U	E/C/U	E/S	C/U	E/S/U	E/U
Interest^b^	Very high	Very high	Very high	High	Medium	Medium
	Complement their RWE strategy (e.g., NICE)	Taxonomy supports local regulators in their role in evaluating, supervision, and safety monitoring of medicines	Taxonomy aligns with their mission to ensure patients get faster access to effective treatments	Maximizes value for money for taxpayers	Advocate for the tools to optimize the use of RWD/RWE for healthcare decisions to improve outcomes, cost savings/efficiencies and sustainability, increasing their political support	Taxonomy provides learning that can be leveraged to improve their understanding of RWD/RWE
	Allows better and more robust RWD/RWE for use in HTA decision making and help agencies learn from the experience of others	Taxonomy can help strengthen public trust in regulatory authorities and their approval processes	Advocate and sponsor financially and facilitate set up of Taxonomy	Increase use of RWE can support HTA decision leading to improved outcomes and efficiencies	Support data governance approach and application of robust methodology	Equipping patient groups with knowledge of RWE in policy/regulation can help to maximize patient involvement, contribution, and application of RW
	Potential user at two levels: (1) to help develop their own guidance and (2) to help individual evaluations of specific technologies through EUNetHTA	EMA: Advocate for the learning healthcare system (DARWIN) – will impact HTA	EFPIA: Having a role in the development of the taxonomy can help deliver on its mission (create collaborative environment to enable innovation and deliver new therapies	Tools for audits/research to assess real outcomes by demographic characteristics to support reducing health inequalities	EU Commission EHDS: Value of healthcare data in decision making to reduce differences in access to medicines across countries/regions and address health inequalities and harmonization of healthcare	Ensure patients are well informed about RWD/RWE so they have trust and confidence in the data they are helping generate
			The taxonomy format aligns with a recent presentation from EFPIA highlighted patient experience case studies	Interest in well-designed RWD/RWE to support commercial deals		

a Roles: E: Endorser/Advocate: a person or organization who publicly supports or recommends the utilization, development, and maintenance of the taxonomy; S: financial sponsor: a person or organization that pays for, or contributes funds to support the maintenance and hosting of the taxonomy; C: Contributor: a person or organization who contributes/donates knowledge/information/expertise to support the maintenance and hosting of the taxonomy; and U: User: a person or organization who utilizes/leverages the taxonomy to support or achieve their own ambition

b Degree of interest: very high, high, medium, and low based on authors’ perceptions.

## Discussion

Two key messages stand out from our collective work (1;3;12–14), and this policy paper. First, transparency, openness, trust, and confidence are key to enhancing RWE use overall in the healthcare system, as well as for HTA purposes specifically. Transparency and openness in the development and use of RWE will support trust and confidence in results, as well as in the data and methodology used. For example, transparency and openness should support the provision of a valid answer to a question where RWE is used, where the limitations are fully understood and can be considered by the decision maker. A transparent and open process can support continuous improvement in how RWE is generated – learning from others and avoiding continually making the same mistakes, keeping abreast of new methods and using the right methods and guides at the right time. Transparency and openness also involves, for example, (i) clearly documenting the limitations of a study when reporting the results, (ii) agencies providing a public explanation for their decisions, and (iii) companies sharing their RWD (but safeguarding when applicable confidential information, similarly to raw data from a RCT not being shared). Within this context, inclusive stakeholder engagement will maximize impact and buy-in, and thus, it is very encouraging to see how the available, albeit limited, HTA bodies’ guidance is addressing these issues, in collaboration and not in isolation. Having clear and transparent international guidance, agreed by consensus, in terms of when to use RWD, how, and for what purposes in the HTA process is something to aspire to. This will require multi-stakeholder cooperation, collaboration and communication across many levels, in a transparent and trusted environment – no single stakeholder will have all the necessary insights and knowledge to cover all aspects to address the current (and future) challenges with RWD acceptance and use.

Second, statements about how new RWD should be used to produce credible RWE should never be made unless the precise question that is to be answered is specified. Whether the data are fit for purpose depends on what the purpose is and what question you are asking.

HTA decision makers and policy makers face many challenges in incorporating evidence into their decisions. While there have been encouraging steps forward, there seems a need to gather real-life examples where RWE has been used, or requested, by HTA/payers, to enable the evaluation of the RWD quality, appraisals assessments, conditional data collection agreements, and their role in decision making (21).

The differences between regulatory and HTA needs and expectations (some justifiable given their different remits) can give rise to the potential contradiction of earlier regulatory approval leading to delayed HTA decision making (3). Joint scientific advice from EMA and HTA bodies to technology developers very early in the process will be critical to align on the evidence to be collected, and the role RWD could play in future assessments. The combination of this advice with official guidelines can improve the efficiency of the process later on during the HTA deliberations – with positive effects for patients and the healthcare system more generally.

At the European regulatory level, the EMA has an important role in supporting the use of RWE. For instance, it has published a Data Quality Framework for medicines regulation (22), providing general considerations on data quality that are relevant for regulatory decision making, definitions for data quality dimensions and subdimensions, as well as their characterization and related metrics. In particular, it sets out definitions, principles, and guidelines that can coherently be applied to a wide range of data sources for the purpose of characterizing and assessing data quality for regulatory decision making. Also, the EHDS (European Health Data Space) could be a game changer, as it is expected to make available a lot of secondary health data with a clear governance framework. Ideally, improved access to data through EHDS can lead to a much welcomed increase in the use of RWD for various use cases, but this would need clear guidelines and frameworks. It would also be important that HTA and payers could determine the studies undertaken under DARWIN EU, expanding its remit beyond regulatory decision making. In addition, the European HTA legislation, which is to be implemented in January 2025, can also be a key driver in boosting the use of RWD for these assessments. Importantly, there needs to be sufficient resources to advise pharmaceutical companies in the evidence requirements, including RWE, as well as ensuring greater alignment across countries in terms of their acceptance of RWE.

With this growing use of RWE, we believe there is support to establish a repository of experience, and we are offering our taxonomy as the starting point of a “live” resource, beyond the material published which is static by nature.

More generally, the RWE framework and this policy article can help to facilitate a common language and terminology, best practice by practitioners when using RWE, and a way to signpost relevant information (e.g., past HTA, pricing, and reimbursement decisions involving the use of RWE) or initiatives that can be accessed quickly to support decisions, together with other criteria deemed important by the decision makers. Our hope is that our RWE framework provides a basis for the construction, by a suitable organization, of a readily accessible repository of HTA reports that will offer learning and case law to guide the collection and presentation of RWDs in the future.

The recent initiatives from HTA bodies certainly align well with the ideal situation outlined in Table 3, and reinforce the need to use both RCTs and RWD. Evidence suggests that appropriately conducted RWD studies and RWE generation could complement RCTs and support HTA decision making (3). The question is not whether one type of evidence is better than the other; the question is how best to use both, to maximize their complementarities. This would, we believe, improve outcomes for patients, and the overall efficiency and sustainability of the healthcare system.

## Conclusions

To conclude, we offer two final reflections.

First, high-quality, fit-for-purpose RWD/RWE can, and should be used more frequently, because it is needed for the creation of evidence packages capable of addressing scientific questions, to inform regulatory, payer, physician, and patient decision making, and to reduce burden on patients, investigators, and healthcare systems. A key element to achieve high-quality credible research is, of course, to seek excellence in scientific rigor – but ensuring the research is then transposed into guidance. However, transparency, reproducibility, and critical assessment of study limitations are also key elements of trust in research findings, and an understanding of previous decisions is central to this. Ultimately, the intended use of RWE, its impact, and its complexity should drive the level of pre-alignment between the different agents when agreeing the RWE to be generated.

Second, multi-stakeholder and cross-geography collaborative partnerships are needed to align on best practices to optimize the evidence that needs to be generated to satisfy all stakeholders’ needs – no single stakeholder will have all the necessary insights and knowledge to cover all aspects to address the current (and future) challenges with RWD acceptance and use, so negotiation and collaboration between the different parties will be needed. Early multi-stakeholder engagement creates opportunities to co-create RWD and RWE, maximize learning via best practice sharing, while minimizing burden for patients.

We welcome engagement with any readers on how the agenda for improvement set out in this policy article can be progressed.
